# Franchising Rabies Vaccine Delivery: The Case of Indian Immunologicals

**DOI:** 10.1371/journal.pntd.0000946

**Published:** 2011-04-26

**Authors:** Hassan Masum, Hima Batavia, Natasha Bhogal, Kim Le, Peter A. Singer

**Affiliations:** 1 McLaughlin-Rotman Centre for Global Health, Toronto, Ontario, Canada; 2 Faculty of Medicine, University of Toronto, Toronto, Ontario, Canada; Instituto Butantan, Brazil

K. V. Balasubramaniam of Indian Immunologicals Ltd. describes their motivation to develop one of the largest vaccine delivery franchises in the world, with over 3,000 locations across India:

*There's an inadequate or poor supply of free vaccines in the public health system, and we don't have universal credibility of the cold chain…so the poorer class near the poverty line has to go and stand in a queue to get the vaccine, to immunize their children. We wanted to bridge that gap. Give the quality, maintain cold chain, affordability, and proper availability throughout the country with a network of qualified people.*

*…We deal with infrastructure that already exists because otherwise we cannot deal with rural costs. It won't be affordable anymore.*

*—K. V. Balasubramaniam, Managing Director, Indian Immunologicals Ltd.*

## Introduction

Roughly 55,000 people worldwide are estimated to die from rabies each year, the majority bitten by unvaccinated stray dogs [1]. In India, on average every two seconds a person is bitten, and every 30 minutes a person dies of rabies [2]. India's National Multicentric Rabies Survey, conducted in 2004 in collaboration with the World Health Organization (WHO), estimated rabies mortality at 20,565 deaths per year; 79% of rabies victims did not receive vaccine treatment [3], [4].

Indian Immunologicals Ltd. (IIL) has contributed to rabies vaccination in India by developing a low-cost rabies vaccine, a national franchise distribution network of 3,000 clinics, and social awareness strategies. IIL has grown into the largest domestic supplier of rabies vaccine, and is now expanding its strategy to other Asian countries.

This paper discusses social, cultural, and economic factors in the development of IIL's rabies vaccine and its distribution system, which will be relevant to readers interested in the development and distribution of treatments for neglected tropical diseases (NTDs). While numerous health delivery franchises have been established in the developing world [5], IIL is to our knowledge one of the largest health delivery franchises for vaccination against an NTD in the world.

Our analysis of IIL's approach draws on a detailed case study conducted between 2005 and 2009, which included semi-structured interviews with key informants, site visits in India, and literature analysis. We conducted interviews with written informed consent with approximately two dozen physicians, franchisors, and IIL personnel. Interviews ranged from 20 minutes to over an hour in length, and all interviews were transcribed and subsequently analyzed. We also analyzed annual reports, background documents from the peer-reviewed literature, news reports, books, government and nongovernmental organization reports, and Web sites. Representatives of IIL were asked to fact-check the case study; the analysis and interpretation is our own. All quotes are from the interviews unless otherwise noted. The case study was approved by the Office of Research Ethics of the University of Toronto.

## How Was an Affordable Rabies Vaccine Developed?

For decades, only nerve tissue rabies vaccine—derived from a sheep or goat—was available in India [2], [3]. Numerous studies showed that severe allergic encephalomyelitis was a possible side effect of the vaccine; however, it was the only affordable option for many developing world populations [6]. The safer and more effective alternative was tissue culture vaccine, endorsed by WHO in the early 2000s as the preferred option [7]. The Indian government discontinued the use of nerve tissue rabies vaccine in 2005 [2], [3], though its occasional availability has still been observed [8].

While tissue culture vaccine options such as Novartis's Rabipur existed in India for high-income populations who could afford to pay, there was a need to develop an option affordable for lower-income communities [7]. Countries such as Thailand had shown trends of declining rabies mortality after making available an affordable vero cell vaccine utilizing the intradermal route [2], [7].

In 1999, the Indian government enlisted IIL to produce a domestic rabies vaccine as part of a larger initiative to domestically develop vaccines against communicable diseases [9]. At the time, IIL specialized in veterinary biologicals. The company was established in 1983 as a wholly owned subsidiary of the public sector National Dairy Development Board of India (NDDB) to support farmers with affordable vaccines. In 1997 and 1998, the company received WHO-GMP and ISO-9001 certification.

As a public–private entity, IIL has a socially oriented charter and a mission of “immunity made affordable,” which translates into a “double bottom line” approach that values both financial and social returns [10], [11]. This gave IIL the freedom to pursue development of a low-cost purified vero cell rabies vaccine. Since this was IIL's first foray into human vaccines, the company launched an independent arm called Human Biologicals.

The vaccine was intentionally produced in-house, explained Dr. K. A. Reddy, IIL Chief General Manager: “A slight delay may be there in the introduction of the product, but we get the technology at a lower price, and as we are going to keep the margins very low, that is going to give us an edge over others to keep our product affordable.” IIL developed the Abhayrab vaccine in two years, with a budget of US$2.5 million.

## How Can a Franchise Model Deliver Vaccines from “Lab to Village”?

By 2001, Abhayrab was approved, after studies done at government facilities. In a subsequent independent study conducted in 2003 by the National Institute for Communicable Diseases, the vaccine was confirmed to be safe and immunogenic [12].

IIL then tackled the challenge of delivering the vaccine affordably. The vaccine's requirement of cold storage, compounded by electricity outages [13] and a five-dose treatment protocol [14], represented substantial delivery challenges.

The majority of IIL's rabies vaccine sales were and still are to the Indian government for use by public hospitals and institutions (see Table 1). However, through a research study, IIL found that when public hospitals were out of stock, patients were sent to retail pharmacies where they often could not afford the first dose. Further, many were unaware of rabies treatment options, reverting to counterfeit medication or traditional practices [15].

**Table 1:** Rabies vaccine sales from 2005 to 2009.

Customer Segment	2004–2005	2005–2006	2006–2007	2007–2008	2008–2009
Indian government	0.92	1.15	2.39	2.77	3.32
Abhay Clinic	0.38	0.57	0.74	0.72	0.73
Abhay Shoppe	0	0.01	0.14	0.39	0.28
*Total*	*1.30*	*1.73*	*3.27*	*3.88*	*4.33*

These findings helped develop a franchise model combining consultation and rabies treatment by targeting physicians in semi-urban and rural areas. The Abhay Clinic was designed to use existing social capital in the form of reputable physicians, leveraging these physicians' office facilities and networks to increase affordability and trust. Support would include educational opportunities, logistics, and marketing initiatives from IIL. In effect, IIL would distribute rabies vaccine in two ways: to the Indian government, and complementarily to the public via an innovative distribution network that we now focus on.

According to the franchise agreement, doctors were required to attend continuing medical education (CME) sessions on animal bite wound treatment and follow-up care, and to maintain proper cold chain. IIL was in turn obligated to pay for these CME sessions, and to maintain cold chain of vaccines until point of delivery to the clinic. The standard of care expected of franchisees was to provide rabies immune globulin for category III bites; it was provided to franchisees at a price point similar to that of the vaccine. However, it was reported that the additional patient cost caused some patients to seek out other doctors who simply gave them the vaccine without rabies immune globulin; franchisee doctors noted this as a point for future public awareness efforts to focus on.

IIL faced difficulty in finding doctors who were both respected within the community and medical profession, and agreeable to only stocking the Abhayrab rabies vaccine. Since profit margins were low, IIL sought socially minded physicians committed to rabies control and eradication: “When we go and discuss with a franchisee the opening of an Abhay Clinic, we very clearly tell him that it's not a very great business—if he wants to do business, he should set up a diagnostic laboratory or make a big nursing home…here he feels he can have more control over vaccination. He doesn't unnecessarily need to send the patient to the retailers,” said Santanu Pal Roy, IIL's Marketing Manager.

According to IIL's management, it took two years to identify the first 500 franchisees. Many were initially apprehensive, and unsure of the benefits of joining a franchise. To encourage enrollment, IIL designed its model to eliminate up-front costs to physicians, and provided franchisees with a refrigerator to maintain cold storage of the vaccine, 30 Abhayrab doses, testing equipment, signage, CME, and social marketing. This cost IIL an estimated Indian rupees (Rs.) 15,000 per franchisee, which would be made back over time. Nevertheless, the right physicians were not always chosen; roughly 10% left or were let go due to behaviors such as stocking rival vaccines in violation of the franchise agreement.

Doctors were sold the vaccine for Rs. 170, and were required to sell it to patients for at most Rs. 220. To meet its mission of “immunity made affordable” while staying economically viable, IIL indicated that it settled for a 15% profit margin to support growth and R&D. Mr. Balasubramaniam claims that “…as an organization, profit maximization is not our motive, but service maximization is, and we also have defined what is a reasonable return on investments.”

## How Were Access Challenges Tackled?

Key requirements to overcoming barriers to accessing health technologies in poor countries have been identified as architecture, availability, affordability, and adoption [16]. Our analysis shows that IIL used social, cultural, and economic factors to meet these requirements.

Its “architecture” combined a public-sector ethos with private-sector efficiencies, giving it latitude to pursue social along with financial imperatives. However, IIL's public ownership might be difficult to replicate in translating the model elsewhere.

By managing both vaccine manufacturing and delivery, IIL eliminated intermediaries in the distribution process, which allowed cost reduction, better supply management, and control over the cold chain. This made the vaccine more “available”. A recent study investigating an oral polio vaccine's cold chain in a rural district of India documented eight levels of storage before reaching the end user, in comparison to IIL's three levels [13]. In 2009, the company reported a field staff of 120, each managing the supply of 30 physicians.

Our analysis showed that IIL's ownership structure, low profit margins, and franchising with physicians contributed to making its vaccine “affordable”. As of 2010, the rabies vaccine was reportedly priced approximately 10% to 30% lower than leading competing vaccines such as Rabipur and Verorab. When physician fees are included, the difference becomes 20% to 40%—a significant amount for lower-income Indians in affordability and in motivating completion of proper vaccination treatment. While pricing is public, detailed analysis of proprietary financial data would be required to reveal the extent to which lower pricing was assisted by IIL's animal health parent enterprise.

Lastly, IIL's understanding of the social factors of rabies has driven “adoption.” Its social marketing campaigns in partnership with schools and community health workers contributed to changing behaviors such as applying chilies, salt, turmeric powder, or lime to clean dog bite wounds [17]. Abhay Clinic doctors were educated on rabies care through sessions on infiltration techniques and rabies immune globulin administration and risks. Franchisees were provided with equipment to perform antibody titer testing, to demonstrate vaccine efficacy. A cause-effect relationship between these interventions and rabies mortality is difficult to establish, but these efforts are likely contributing to increased appropriate rabies care.

By overcoming vaccine distribution challenges, IIL delivered over 4 million (M) rabies doses through its distribution channels in 2009, including the Indian government (3.32 M doses), Abhay Clinics (0.73 M doses), and Abhay Shoppes (0.28 M doses). (See Table 1 for 5 years of data.) Revenues from the vaccine were Rs. 843 M in the 2009 reporting year, up from Rs. 636 M the previous year; see [10], [11] for further financial data.

## How Is Indian Immunologicals Evolving Its Approach?

Since building its Abhay Clinic network, IIL has evolved its approach in three ways. First, in 2006 it developed “Abhay Shoppes” for delivery of small quantities of rabies vaccine to doctors—an “on-call” service aimed at non-franchisee doctors who did not want to commit exclusively to IIL's vaccine. As of 2009, IIL had 300 Abhay Shoppes supplying rabies vaccine to roughly 100 physicians each. However, some Shoppe owners have expressed dissatisfaction with their income and some Abhay Clinic franchisees are concerned about encroachment; vaccine delivery through Shoppes dropped in 2009 from the previous year.

Second, IIL is replicating its Abhay model in other countries. Since 2007 in the Philippines, the model has been modified to meet local conditions. In contrast to Abhay Clinics, the Philippines model involves partnering with government facilities, creating adjunct “Animal Bite Treatment Centers”, and charging patients per (intradermal) shot. This has helped patients spread cost across several visits. Expansion to other Asian countries is under consideration.

Finally, IIL has expanded its human vaccine line, including AbhayTox for tetanus, AbhayM for measles, and AbhayTag for diphtheria, pertussis, and tetanus. It has started supplying these to Abhay Clinics, with plans to transition the franchises into “One Stop Vaccination Centers”. However, since many vaccines for these conditions are available for free at government hospitals, or are administered to children by pediatricians [18], only some general practitioners in IIL's network have been receptive to buying the additional vaccines. The company has also been collaborating with the non-profit Program for Appropriate Technology in Health since 2003, in a multi-partner initiative to optimize the heat- and freeze-stability of several types of vaccines [19].

## Conclusion

Our case study reveals that social, cultural, and economic factors were critical to IIL's franchise network reaching its current scale. Social and cultural factors included the income and geographical distribution of its target population; rabies awareness in both adults and children; community-based reputation of physicians; and incentives for franchisees.

Economic factors included eliminating middlemen for both cost savings and improved cold chain maintenance; leveraging existing physician infrastructure to reduce distribution network expenses; minimizing up-front payments for franchising physicians; tapping previous animal vaccine development expertise; and an unusual public–private ownership structure that has led to a focus on social impact along with profits.

While the Abhay approach has demonstrably scaled across India, its health impact to date is difficult to quantify, and this is an area for future research. Ultimately, the eradication of rabies requires controlling the spread within animals, and further educating patients [20].

IIL has built one of the largest NTD vaccine delivery franchises in the world, with almost 3,000 locations. This vaccine delivery and awareness model may hold lessons for future NTD efforts.

**Learning Points**

- Leveraging existing physicians through a franchise model can cut costs to provide affordable treatment options and speed adoption, especially when the physicians selected are trusted community leaders.
- Programs to educate the community and develop trust can be effective social and cultural components of large-scale health care delivery strategies.
- Publicly owned organizations with private-sector efficiencies can combine the best of both worlds by manufacturing and delivering neglected disease treatments and complementing strained public health care systems. The ownership structure of IIL has the flexibility to consider social factors in decision-making.
- Development of health solutions by organizations based in developing countries can have significant advantages—including commitment and ability to create affordable products, physical proximity to the market, and appreciation of cultural nuances and end-user behavior.
